# MEDIAL-LATERAL POSTURAL SWAY INCREASE AMONG COMMUNITY-DWELLING OLDER ADULTS WITH SELF-REPORTED ANXIETY

**DOI:** 10.1093/geroni/igae098.4024

**Published:** 2024-12-31

**Authors:** Jethro Raphael Suarez, Amber Blount, Kworweinski Lafontant, Nichole Lighthall, Helen Huang, Joon-Hyuk Park, Ladda Thiamwong

**Affiliations:** University of Central Florida; University of Central Florida, Orlando, Florida, United States; University of Central Florida, Orlando, Florida, United States; University of Central Florida, Orlando, Florida, United States; University of Central Florida, Orlando, Florida, United States; University of Central Florida, Orlando, Florida, United States; University of Central Florida, Orlando, Florida, United States

## Abstract

Anxiety is a prevalent and concerning issue for older adults. Previous research has shown that anxiety negatively affects overall balance, but the effect of self-reported anxiety on postural sway direction for older adults is unclear. This study aimed to compare sway direction between community-dwelling older adults that were categorized as having moderate-to-high anxiety and those that had low-to-no anxiety. The BTrackS Balance System (BBS), a stationary and simple balance assessment instrument, was used for measuring ranges of sway in the medial-lateral and anterior-posterior directions during a non-visual static balance test. The Geriatric Anxiety Inventory Short Form (GAI-SF) is a self-reported assessment of anxiety and was used for categorization. We cross-sectionally compared sway direction from 170 community-dwelling older adults aged 61 to 91 (152 women, mean age = 74.3 ± 7.1 years) who completed a BBS assessment and completed the GAI-SF using Mann-Whitney U tests. A total of 133 older adults were categorized as having low-to-no anxiety (GAI-SF ≤ 2), while 37 were categorized as having moderate-to-high anxiety (GAI-SF ≥ 3). The groups significantly differed in the medial-lateral direction (p = 0.03), but not the anterior-posterior direction (p = 0.14). These results show that older adults with increased self-reported anxiety may move in the medial-lateral direction more than those with less self-reported anxiety. However, these results may be limited by other factors (e.g., past injuries, depression). Future research should consider such factors among older adults that show symptoms of anxiety.

